# The role of the FT3/FT4 ratio in predicting remission and relapse in pediatric Graves’ disease

**DOI:** 10.1007/s00431-026-06808-7

**Published:** 2026-03-10

**Authors:** Leyla Kara, Ulku Gul Siraz, Dilek Cicek, Emre Sarikaya, Ebru Gok, Ugur Berber, Mustafa Kendirci, Selim Kurtoğlu, Nihal Hatipoglu

**Affiliations:** Department of Pediatric Endocrinology, Erciyes University Faculty of Medicine Hospitals, Kayseri, Turkey

**Keywords:** Graves’ disease, Hyperthyroidism, FT3/FT4 ratio, Thyrotropin receptor antibodies (TRAb), Remission, Relapse, Antithyroid drugs

## Abstract

Long-term antithyroid drug (ATD) therapy is often required in pediatric Graves’ disease, yet adherence can be challenging. Therefore, identifying reliable predictors of remission and relapse is crucial for optimizing disease management. This study aimed to evaluate the role of the baseline FT3/FT4 ratio in predicting remission and relapse, alongside other potential markers, including TRAb levels, thyroid volume, and body mass index (BMI). This retrospective, single-center study included pediatric patients diagnosed with Graves’ disease and treated with ATDs. Age, sex, BMI, thyroid volume, TRAb, FT3, and FT4 levels were recorded at diagnosis, and the FT3/FT4 ratio was calculated. Remission was defined as maintaining euthyroidism for at least 12 months after discontinuation of ATD therapy, whereas relapse was defined as the recurrence of hyperthyroidism following remission. A total of 55 patients were evaluated. The remission rate was 43.6%, and among those who achieved remission, the relapse rate was 8.3%. No significant association was found between remission and age, sex, BMI, ATD duration, or the FT3/FT4 ratio. However, remission was significantly more likely in patients with TRAb levels < 10 IU/L (*p* < 0.05). Additionally, an inverse relationship was observed between thyroid volume and the likelihood of remission. Although the baseline FT3/FT4 ratio did not predict remission, it was significantly higher in patients who experienced relapse. An FT3/FT4 ratio > 0.54 pmol/pmol predicted relapse with 75% sensitivity and 98% specificity (AUC = 0.842; *p* = 0.048).

*Conclusion*: The baseline FT3/FT4 ratio demonstrated limited value in predicting remission but proved to be a strong indicator of relapse risk in pediatric Graves’ disease. Additionally, lower TRAb levels (< 10 IU/L) and smaller thyroid volumes were associated with higher remission rates. Together, these findings suggest that incorporating the FT3/FT4 ratio, TRAb levels, and thyroid volume into routine assessment may enhance risk stratification and support more personalized treatment approaches for pediatric Graves’ disease.

**Table:** 

**What is Known:**
• *Pediatric Graves’ disease often requires long-term antithyroid drug therapy, and remission rates are variable; TRAb levels and thyroid volume are recognized predictors of remission.*
**What is New:**
• *The baseline FT3/FT4 ratio has limited value in predicting remission but may serve as a strong predictor of relapse, with a ratio 0.54 pmol/pmol showing high specifi city for relapse in pediatric Graves’ disease.*

## Introduction

Graves’ disease is the most common cause of acquired autoimmune hyperthyroidism in childhood, and it is typically characterized by elevated levels of free triiodothyronine (FT3) and free thyroxine (FT4) accompanied by suppressed thyroid-stimulating hormone (TSH) levels [1]. In children, remission rates are generally reported to range between 20 and 30%, and the majority of patients require long-term antithyroid drug (ATD) therapy. However, predicting remission or relapse during treatment remains a major clinical challenge. Although several prognostic factors—such as thyroid-stimulating hormone receptor antibody (TRAb) levels, goiter volume, and treatment duration—have been identified, pediatric data on biochemical predictors are still quite limited [2]. Recent evidence suggests that the free T3 to free T4 (FT3/FT4) ratio may have utility in the evaluation of pediatric Graves’ disease. Shimura et al. demonstrated that pediatric GD patients had significantly higher FT3/FT4 ratios compared to non-GD controls, supporting the potential role of this ratio as a biochemical marker in GD assessment [3]. Although direct evidence linking the FT3/FT4 ratio to long-term relapse or remission outcomes is limited, some cohort studies indicate that higher thyroid hormone levels and related markers may be associated with more aggressive disease or lower remission likelihood.

This study aimed to evaluate the predictive value of the FT3/FT4 ratio at diagnosis for sustained remission and relapse in children diagnosed with Graves’ disease. In this context, other clinical and biochemical parameters, including body mass index (BMI), TRAb levels, and thyroid volume, were also assessed. Additionally, treatment duration and the potential for establishing a practical biomarker to guide individualized management were investigated.

## Methods

### Study design and population

This retrospective study included 55 pediatric patients diagnosed with Graves’ disease and followed up at the Pediatric Endocrinology Clinic of Erciyes University Faculty of Medicine between March 2010 and February 2022. Patients aged 1 to 18 years, diagnosed with Graves’ disease based on clinical findings, suppressed TSH, elevated FT4 and/or FT3 levels, and positive TRAb, who had received antithyroid drug (ATD) therapy for at least 1 year and had regular follow-up for at least 1 year, were included. Patients who underwent radioactive iodine therapy (RAI) or thyroidectomy were also included.

Patients with non-autoimmune causes of hyperthyroidism (e.g., toxic nodular goiter, subacute thyroiditis), those with concomitant genetic or systemic disorders (e.g., Down syndrome, Turner syndrome), patients with insufficient clinical or laboratory data, and those with a follow-up duration shorter than 12 months were excluded.

### Definition of remission

Remission was defined as the maintenance of clinical and biochemical euthyroidism for at least 1 year after discontinuation of ATD therapy, without any relapse during this period [2]. Relapse was defined as an increase in FT4 or FT3 levels accompanied by suppressed serum TSH following a minimum of 12 months of adequate treatment, after dose reduction or discontinuation of ATD therapy [4]. Three patients who underwent surgery or radioactive iodine (RAI) therapy shortly after the initiation of ATD treatment were excluded from the remission and non-remission group comparisons, as they did not receive an adequate duration of therapy, and their remission potential could not be evaluated. Initial methimazole doses were adjusted by weight and disease severity, with titration according to thyroid function tests. ATD were discontinued when patients were clinically euthyroid and thyroid hormones normalized. TRAb levels were measured at cessation; some patients had low-level positivity. In line with current guidelines, ATD could be stopped even if TRAb remained mildly positive, as long-term remission depends more on treatment duration and TRAb trends than on absolute TRAb values [5].

### Data collection

Patient records were retrospectively reviewed to collect data on age, sex, body weight, weight standard deviation score (SDS), height SDS, body mass index (BMI) SDS, pubertal status (according to Tanner staging), family history of autoimmune thyroid disease, and the presence of ophthalmopathy and goiter.

Laboratory parameters obtained at diagnosis included free triiodothyronine (FT3; reference range 3.53–6.45 pmol/L), free thyroxine (FT4; reference range 11.45–28.31 pmol/L), FT3/FT4 ratio, thyroid-stimulating hormone receptor antibody (TRAb; 0–1.7 IU/L), antithyroid peroxidase antibody (anti-TPO; 0–9 IU/mL), and antithyroglobulin antibody (anti-TG; 0–4 IU/mL).

Thyroid gland volume and volume SDS values were calculated based on thyroid ultrasonography findings using the TPEDS Metrics software (ÇEDD Solution) [5]. Additionally, the time to normalization of TSH, FT3, FT4, and TRAb levels was recorded (TRAb levels were measured at diagnosis and at the end of at least the first year of treatment). Information regarding the type of antithyroid drug (ATD) used, duration of treatment, and total follow-up period was also collected. The FT3/FT4 ratio was calculated by dividing the FT3 value by the FT4 value (pmol/L ÷ pmol/L).

### Statistical analysis

Data were analyzed using SPSS software (IBM SPSS Statistics 25.0). The distribution of continuous variables was assessed by the Shapiro–Wilk test. Normally distributed data were presented as mean ± standard deviation (SD), while non-normally distributed data were expressed as median and interquartile range (IQR). For comparisons between two groups, the Student *t*-test was used for normally distributed variables, and the Mann–Whitney *U* test was used for non-normally distributed variables. Categorical variables were compared using the chi-square test. The predictive performance of the FT3/FT4 ratio for remission was evaluated by receiver operating characteristic (ROC) curve analysis. Patients in the remission group were considered positive, while those not achieving remission were considered negative. Area under the curve (AUC), sensitivity, and specificity values were calculated. The same analysis was performed to evaluate the predictive ability of the FT3/FT4 ratio at diagnosis for relapse. A *p*-value < 0.05 was considered statistically significant. This study was approved by the Erciyes University Clinical Research Ethics Committee (Ref. No: 2022/629, 14.09.2022).

## Results

**Fig. 1:**
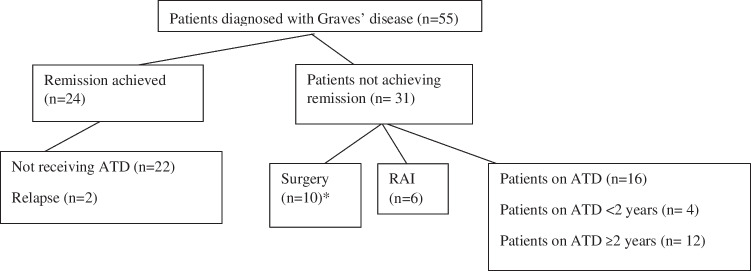
Flow diagram illustrating the clinical outcomes and treatment modalities of patients diagnosed with Graves’ disease (*n* = 55). Patients were categorized according to remission status, relapse, and treatment approach, including antithyroid drug (ATD) therapy, radioactive iodine (RAI), and surgery

The study cohort consisted of 55 pediatric patients with a mean age of 12.85 ± 3.81 years, of whom 76.4% were female. Remission was achieved in 24 patients (43.6%) following a mean duration of 3.8 years (range 2.41–4.45 years) of antithyroid drug (ATD) therapy. Among these, two patients (8.3%) experienced relapse at 1.25 and 2.08 years after remission, respectively. The remaining 31 patients (56.4%) did not attain remission despite a mean treatment duration of 3.54 years (range 2.36–5.48 years) (*p* = 0.725) (Fig. 1).

Radioactive iodine (RAI) therapy was administered to six patients after a mean of 2.37 ± 1 years of ATD treatment. In one patient, RAI was initiated 0.75 years after diagnosis due to drug allergy; however, relapse occurred 0.66 years after RAI, necessitating total thyroidectomy.

In total, ten patients who either relapsed or failed to achieve remission underwent total thyroidectomy after a mean of 5.03 ± 3.23 years of follow-up. Additionally, two patients underwent primary surgical intervention owing to the presence of thyroid nodules. Histopathological examination revealed papillary thyroid carcinoma in two cases (3%).

The demographic and clinical features of the entire cohort are summarized in Table 1. For further analysis, patients were stratified into two groups based on their ATD treatment outcomes: those achieving sustained remission and those who did not (Table 2). Three patients who underwent early RAI or surgery were excluded due to unassessable remission status. Similarly, two patients who relapsed after achieving remission were excluded from the analysis of sustained remission.

**Table 1:** Baseline demographic, clinical, and laboratory characteristics of the patients

Parameter	Mean ± SD/median (IQR) or presence (%)
Age (years)	12.85 ± 3.81
Follow-up duration (years)	3.60 (2.51–5.66)
Male sex (%)	23.6%
Body weight SDS	− 0.25 ± 1.26
Height SDS	− 0.03 ± 1.21
BMI SDS	− 0.33 ± 1.23
Pubertal (%)	83.6%
Family history of autoimmune thyroid disease (%)	18.2%
Presence of goiter (%)	83.6%
Presence of ophthalmopathy (%)	65.6%
FT3 (pmol/L)	20.58 ± 11.25
FT4 (pmol/L)	53.41 ± 28.61
FT3/FT4 ratio (pmol/L/pmol/L)	0.42 (0.35–0.48)
TRAb (IU/L)	17.23 ± 15.94
TRAb < 10 IU/L (%)	46%
Thyroid volume (mL)	12.80 (7.58–22.00)
Thyroid volume SDS	5.11 (1.78–11.41)
Time to FT3 normalization (weeks)	2 (1–5)
Time to FT4 normalization (weeks)	2 (1–4)
Time to TSH normalization (weeks)	4 (2–10)
Time to TRAb normalization (years)	2.73 ± 1.76
Patients with TRAb normalization (%)	32%
Duration of drug treatment (years)	3.47 (2.32–4.64)

Data are presented as mean ± standard deviation or median (IQR), and percentages represent the prevalence in the patient group

*SDS* standard deviation score, *BMI* body mass index, *FT3* free triiodothyronine, *FT4* free thyroxine, *TRAb* TSH receptor antibody, *Anti-TPO* anti-thyroid peroxidase antibody, *Anti-TG* anti-thyroglobulin antibody, *IQR* interquartile range

**Table 2:** Comparison of clinical characteristics between patients with and without remission

Parameter	Remission (*n* = 22)	No remission (*n* = 28)	*p*-value
Age (years)	12.66 ± 3.54	13.09 ± 3.76	0.681
Follow-up duration (years)	4.21 ± 1.69	4.89 ± 3.34	0.386
Male sex (%)	18%	28%	0.393
Body weight SDS	− 0.55 ± 1.14	− 0.14 ± 1.05	0.203
Height SDS	− 0.38 ± 1.34	0.20 ± 0.97	0.075
BMI SDS	− 0.36 ± 1.07	− 0.42 ± 1.25	0.866
Pubertal status (%)	81%	85%	0.709
Family history of autoimmune thyroid disease (%)	22%	17%	0.669
Presence of goiter (%)	79%	89%	0.752
Presence of ophthalmopathy (%)	63%	67%	0.754
FT3 (pmol/L)	19.89 (10.44–25.57)	27.14 (17.35–35.09)	0.095
FT4 (pmol/L)	48.84 (35.26–69.30)	56.94 (44.04–91.05)	0.143
FT3/FT4 ratio (pmol/L/pmol/L)	0.39 (0.36–0.44)	0.42 (0.35–0.50)	0.369
TRAb (IU/L)	14.68 ± 18.55	19.23 ± 13.56	0.322
TRAb < 10 IU/L (%)	63%	32%	0.027
Thyroid volume (mL)	13.19 ± 7.93	18.05 ± 13.42	0.143
Thyroid volume SDS	4.77 ± 4.37	6.69 ± 6.89	0.287
Time to FT3 normalization (weeks)	2.25 (0.92–5.25)	2.00 (1.37–4.00)	0.669
Time to FT4 normalization (weeks)	2.25 (1.00–4.00)	2.00 (1.00–4.00)	0.100
Time to TSH normalization (weeks)	4.50 (2.00–6.50)	2.75 (1.37–12.25)	0.648
Time to TRAb normalization (years)	2.40 ± 1.37	3.54 ± 2.46	0.234
Patients with TRAb normalization (%)	54%	17%	0.007
Duration of drug treatment (years)	3.51 ± 1.13	4.21 ± 2.94	0.300

Data are presented as mean ± standard deviation or median (IQR). Percentages represent the frequency within each group

*SDS* standard deviation score, *BMI* body mass index, *FT3* free triiodothyronine, *FT4* free thyroxine, *TRAb* TSH receptor antibody, *Anti-TPO* anti-thyroid peroxidase antibody, *Anti-TG* anti-thyroglobulin antibody, *IQR* interquartile range

As a result, 22 patients (44%) constituted the sustained remission group, whereas 28 patients (56%) comprised the non-remission group. No significant differences were observed between the groups regarding baseline FT3, FT4, TRAb levels, thyroid volume, or time to biochemical normalization (*p* > 0.05). Nevertheless, the proportion of patients with TRAb < 10 IU/L and those achieving TRAb normalization was significantly higher in the remission group compared to the non-remission group (63% vs. 32%, *p* = 0.027; 54% vs. 17%, *p* = 0.007, respectively).

To identify predictors of remission, a multivariate logistic regression analysis was performed including age, sex, BMI, FT3/FT4 ratio, TRAb level, thyroid volume, and duration of antithyroid drug (ATD) therapy. The overall model demonstrated good fit according to the Hosmer–Lemeshow test (*p* = 0.704) and explained 21.3% of the variance in remission outcomes (Nagelkerke *R*^2^ = 0.213). Although none of the included variables were statistically significant (all *p* > 0.05), female sex (OR = 0.346, 95% CI 0.063–1.904, *p* = 0.223) and ATD treatment duration (OR = 0.682, 95% CI 0.432–1.079, *p* = 0.102) showed trends toward a decreased likelihood of remission (Table 3).

**Table 3:** Logistic regression analysis of factors predicting remission

Variable	*B*	Std. error	Wald	*p*-value	Exp(B)	95% CI for Exp(B)
Constant	3.444	2.545	1.831	0.176	31.299	–
Age (years)	0.000	0.131	0.000	0.999	1.000	0.773–1.292
Sex (female = 1)	–1.061	0.870	1.488	0.223	0.346	0.063–1.904
BMI (kg/m^2^)	–0.021	0.108	0.038	0.845	0.979	0.792–1.211
FT3/FT4 ratio (pmol/L/pmol/L)	–0.088	0.349	0.063	0.802	0.916	0.462–1.816
TRAb level (IU/L)	–0.009	0.023	0.155	0.694	0.991	0.948–1.036
Thyroid volume (mL)	–0.068	0.043	2.515	0.113	0.935	0.860–1.016
Duration of antithyroid drug use (years)	–0.382	0.234	2.676	0.102	0.682	0.432–1.079

Model fit statistics: Hosmer–Lemeshow test *p* = 0.704, Nagelkerke *R*^2^ = 0.213, *n* = 50. Statistical significance was accepted at *p* < 0.05

*B* regression coefficient, *Std. error* standard error, *Exp(B)* odds ratio, *CI* 95% confidence interval, *BMI* body mass index, *TRAb* TSH receptor antibody, *ATD* antithyroid drug

Receiver operating characteristic (ROC) curve analysis was conducted to evaluate the discriminative ability of clinical parameters in predicting remission. Among the tested variables, only TRAb levels were found to be statistically significant (AUC = 0.321, *p* = 0.034). However, the AUC value below 0.5 indicated that lower TRAb levels were inversely associated with remission. A TRAb threshold of < 10.45 IU/L was identified as the optimal cutoff, yielding a sensitivity of 45% and specificity of 33%.

For age, BMI, FT3/FT4 ratio, thyroid volume, and ATD treatment duration, AUC values were close to or below 0.5 and did not reach statistical significance (*p* > 0.05). These findings suggest that the evaluated clinical and biochemical parameters have limited discriminatory power in accurately predicting remission (Table 4).

**Table 4:** ROC analysis results of clinical parameters predicting remission

Parameter	AUC	Std. error	*p*-value	95% CI for AUC
Age (years)	0.465	0.084	0.679	0.300–0.630
BMI (kg/m^2^)	0.525	0.084	0.764	0.360–0.691
FT3/FT4 ratio	0.411	0.084	0.291	0.247–0.575
TRAb level (IU/L)	0.321	0.079	0.034	0.166–0.476
Thyroid volume (mL)	0.425	0.084	0.374	0.261–0.589
Duration of ATD use (years)	0.451	0.085	0.562	0.284–0.618

*P*-value < 0.05 was considered statistically significant

*AUC* area under the curve, *Std. error* standard error, *CI* 95% confidence interval for AUC, *BMI* body mass index, *TRAb* TSH receptor antibody, *ATD* antithyroid drug

**Fig. 2:**
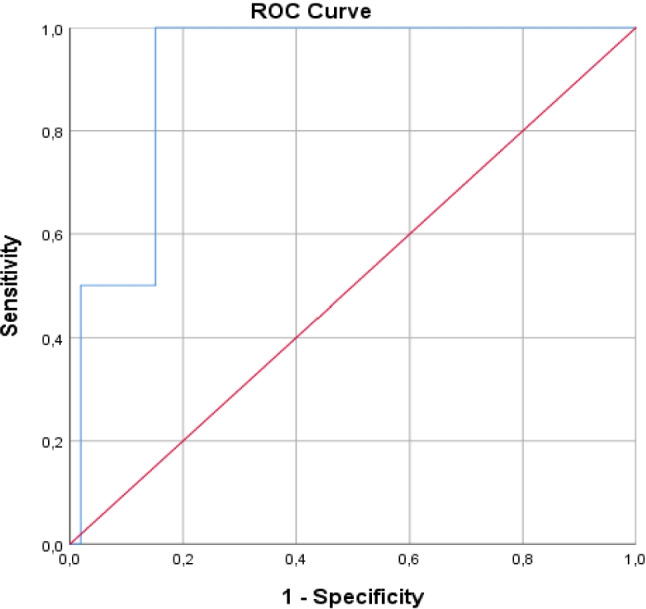
Receiver operating characteristic (ROC) curve analysis of the FT3/FT4 ratio for predicting relapse. The area under the curve (AUC) was 0.842. A cutoff value of 0.54 yielded a sensitivity of 98% and a specificity of 75%

In contrast, the FT3/FT4 ratio demonstrated a significant ability to predict relapse (AUC = 0.842, *p* = 0.024). A cutoff value of 0.54 pmol/L per pmol/L provided a sensitivity of 98% and specificity of 75%, suggesting that the FT3/FT4 ratio may serve as a useful biomarker for relapse prediction. Nevertheless, because the analysis was based on only two relapse events, the statistical power is limited, and these findings should be interpreted with caution (Fig. 2).

## Discussion

This study aimed to evaluate clinical and biochemical markers associated with remission and relapse in pediatric patients with Graves’ disease receiving antithyroid drug (ATD) therapy. Pediatric Graves’ disease often presents with different clinical features and treatment responses compared to adults; therefore, identifying reliable prognostic parameters is of great clinical importance. Although various factors have been proposed in the literature to predict remission and relapse, data specific to the pediatric population remain limited and results are heterogeneous. We acknowledge that our relatively small sample size may contribute to this heterogeneity. Nevertheless, our study provides additional information from a long-term follow-up cohort (mean 3.66 years) and emphasizes that relapse can occur beyond the first year of remission. While our findings supplement existing knowledge, they should be interpreted with caution, and larger, multicenter studies are needed to identify robust predictors of remission and relapse in pediatric patients.

In our study, the majority of pediatric patients with Graves’ disease were found to be female and in the pubertal period. This finding is consistent with existing literature indicating that the disease is more frequently observed in post-pubertal girls. Indeed, in the multicenter cohort study by Esen et al., it was reported that more than 75% of the cases were female and that the mean age at diagnosis predominantly ranged between 11 and 14 years [6]. Furthermore, elevated TRAb levels along with the presence of anti-TPO and anti-TG antibodies support the autoimmune nature of the disease. The frequencies of goiter and ophthalmopathy observed in our cohort are also in line with previous reports. In addition, the high thyroid volume and volume SDS values are consistent with earlier studies indicating that pediatric Graves’ disease is frequently characterized by marked thyroid enlargement [7].

In adults, remission rates following antithyroid drug (ATD) therapy have been reported to range between 40 and 50%, while relapse rates may reach 30 to 70% [8, 9]. In the study by Magri et al., a remission rate of 54.1% and a relapse rate of 11.2% were observed over a 5-year follow-up [10]. In pediatric patients, remission rates have been shown to increase with longer treatment duration, reaching approximately 18% by the fourth year and 50% by the fifth year of follow-up[11]. However, the incidence of relapse after ATD discontinuation has been reported to vary widely, from 3 to 47% [4].

In the present study, remission was defined as maintaining a euthyroid state for at least 12 months after discontinuation of ATD therapy. Using this definition, 43.6% of our pediatric Graves’ disease patients achieved remission. Importantly, it is well recognized that in children, a substantial proportion may experience relapse beyond the first year, with reported rates ranging from 20 to 40% [12]. In our cohort, patients were followed for a mean duration of 3.66 years, and relapse occurred in 8.3% of those who initially achieved remission, specifically beyond the first 12 months. These findings emphasize the importance of long-term follow-up in pediatric Graves’ disease, even after apparent remission, and are consistent with previous literature.

No significant associations were found between remission and age, sex, BMI, FT3/FT4 ratio, or duration of ATD use. Similarly, a prospective study on pediatric Graves’ disease reported that these parameters were not predictive of remission. In this 2008 study, only early achievement of euthyroidism, low baseline T3 levels, and older age were identified as factors associated with remission after 2 years of antithyroid drug therapy; however, no significant relationships were observed with age, sex, BMI, or thyroid volume [2]. Consistent with previous findings, our study also highlights the limited predictive value of certain parameters for remission. However, unlike some reports in the literature, we found that increased TRAb levels and thyroid volume were inversely associated with remission. Furthermore, only 8.3% of the 24 patients who achieved remission experienced relapse, which is relatively low compared to the 3 to 47% relapse rates reported in the literature [2, 4]. The relatively long duration of ATD therapy administered at our center (mean ~ 3.8 years) may have contributed to more sustained remission. A prospective study conducted in 2012 emphasized that continuous ATD treatment for 8–10 years could increase remission rates, highlighting the importance of close monitoring of patients during this period [13].

Studies have reported conflicting results regarding the role of the FT3/FT4 ratio in predicting disease severity and response to treatment. A subset of pediatric Graves’ disease patients characterized by T3 dominance (T3-P-GD) has been described, with these patients reportedly being younger, exhibiting higher TRAb levels, and experiencing more severe hyperthyroidism. Furthermore, it has been demonstrated that these patients maintain a higher FT3/FT4 ratio throughout treatment and require higher doses of antithyroid drugs [14]. In our study, the strong predictive power of the FT3/FT4 ratio for relapse supports the notion that a T3-dominant response pattern is associated with a more aggressive disease course. In a study conducted in adult patients with Graves’ disease, the FT3/FT4 ratio was found to be positively correlated with both TRAb levels and thyroid volume, suggesting that elevated FT3 levels are associated with disease severity and risk of relapse [15]. In a retrospective study by Bayramoğlu et al., male sex and elevated FT4 levels at diagnosis were found to be associated with relapse, whereas a high FT3/FT4 ratio and longer duration of methimazole treatment were significantly associated with remission [16]. However, in our study, no significant associations were found between remission and parameters such as age, sex, BMI, FT3/FT4 ratio, or duration of ATD therapy. These variations may be attributed to differences in study populations, treatment protocols, definition criteria, and follow-up durations.

Another study reported that the FT3/FT4 ratio at diagnosis was associated with failure to achieve remission and relapse. Additionally, increased thyroid volume has been linked to poor prognostic outcomes [17]. Consistent with similar studies, our findings also demonstrated a significant negative association between increased thyroid volume and remission status. Although the FT3/FT4 ratio at diagnosis was not a significant predictor of remission, its high sensitivity and specificity in predicting relapse is noteworthy. These results suggest that the FT3/FT4 ratio may serve as an important biomarker for evaluating disease course and relapse risk in Graves’ disease. It is believed that this ratio could aid in assessing a patient’s relapse risk profile before the discontinuation of long-term ATD therapy. FT3/FT4 ratio values above 0.54 pmol/L/pmol/L may be used to identify high-risk groups.

While TRAb levels have been reported in the literature as a potential biomarker for treatment response and remission prediction, their association appears more variable and inconsistent within the pediatric population [18, 19]. Especially, TRAb negativity has been proposed as a criterion for the discontinuation of antithyroid drug therapy [20]. In our study, a non-significant negative correlation was observed between TRAb levels and remission. However, it is noteworthy that the majority of patients whose TRAb levels normalized belonged to the remission group. This finding supports the potential utility of TRAb levels in predicting remission. Additionally, patients with TRAb levels below 10 IU/L had a significantly higher remission rate in our cohort. This observation is consistent with previous studies indicating that TRAb levels serve as a prognostic marker for achieving remission. In a prospective study by Karmisholt et al., 63% of patients with baseline TRAb levels < 10 IU/L achieved remission, compared to 39% of those with TRAb levels ≥ 10 IU/L, with the former group reaching remission approximately 5 months earlier on average [21].

## Limitations

Although the sample size of our study is relatively small and it was conducted at a single center, the findings highlight the potential value of the FT3/FT4 ratio in predicting relapse in pediatric Graves’ disease. Nevertheless, larger multicenter prospective studies are needed to validate these results and confirm their reliability and generalizability. The relatively small sample size of our study limits its statistical power, increasing the risk of a type II error.

## Conclusion

Our results indicate that a higher FT3/FT4 ratio at diagnosis, together with larger thyroid volume and elevated TRAb levels, reflects a more severe disease presentation and is associated with an increased risk of relapse in pediatric Graves’ disease. Notably, the FT3/FT4 ratio appears to be more useful in identifying patients at higher risk of future relapse rather than reliably predicting remission. Therefore, this parameter may serve as an adjunctive tool for clinical risk stratification and individualized management, although it does not fully address all challenges in the treatment of pediatric Graves’ disease.

## Data Availability

The data supporting the findings of this study are available from the corresponding author upon reasonable request.
